# Endothelial cell damage in patients with acute graft versus host disease receiving treatment with extracorporeal photopheresis

**DOI:** 10.3389/fimmu.2026.1852600

**Published:** 2026-06-15

**Authors:** Julia Martinez-Sanchez, Paola Charry, Ana Belén Moreno-Castaño, Alex Ramos, Sergi Torramade-Moix, Helena Ventosa-Capell, Marta Palomo, Olaf Penack, María Queralt Salas, María Suárez-Lledó, Francesc Fernández-Avilés, Carmen Martínez, Laura Rosiñol, Montserrat Rovira, Enric Carreras, Miquel Lozano, Gines Escolar, Joan Cid, Maribel Diaz-Ricart

**Affiliations:** 1 Hemostasis and Erythropathology Laboratory, Hematopathology, Department of Pathology, Centre de Diagnòstic Biomèdic (CDB), Hospital Clínic de Barcelona, Institut d’Investigacions Biomèdiques August Pi i Sunyer (IDIBAPS), Universitat de Barcelona, Barcelona, Spain; 2 Barcelona Endothelium Team, Barcelona, Spain; 3 Apheresis and Cellular Therapy Unit, Hemotherapy and Hemostasis Department, Institut del Càncer i Malalties de la Sang (ICAMS), Hospital Clínic de Barcelona, IDIBAPS, Universitat de Barcelona, Barcelona, Spain; 4 Medical Intensive Care Unit, Hospital Clínic de Barcelona, Barcelona, Spain; 5 Hematology External Quality Assessment Laboratory, CDB, Hospital Clínic de Barcelona, Barcelona, Spain; 6 Hematology, Oncology and Tumorimmunology Department, Charité-Universitätsmedizin Berlin, Freie Universität Berlin and Humboldt-Universität zu Berlin, Berlin, Germany; 7 Hematopoietic Transplantation Unit, Hematology Department, ICAMS, Hospital Clínic de Barcelona, IDIBAPS, Universitat de Barcelona, Barcelona, Spain; 8 Fundació Josep Carreras Contra la Leucèmia, Barcelona, Spain

**Keywords:** endothelial damage, endothelium, extracorporeal photopheresis, soluble biomarkers, steroid-refractory aGVHD

## Abstract

**Introduction:**

Extracorporeal photopheresis (ECP) is a safe, effective treatment for steroid-refractory acute GVHD (SR-aGVHD). Endothelial damage is a pathological substrate of aGVHD.

**Methods:**

Endothelial damage biomarkers were measured in SR-aGVHD patients' plasma before (PRE) and 1-month after initiating ECP as second-line therapy to explore differences by treatment response. ECP-treated SR-aGVHD patients (n=35) were classified into good (GR; n=18) and poor (PR; n=17) responders. Endothelial activation biomarkers (soluble Vascular Cell Adhesion Molecule-1, sVCAM-1; von Willebrand Factor, VWF; thrombomodulin, TM; soluble TNF receptor 1, sTNFR1; angiopoietin 2; ANG2); GVHD markers (suppression tumorigenicity 2, ST2; regenerating islet-derived 3-alpha, REG3alpha; T-cell immunoglobulinmucin-3, TIM3); soluble C5b9 (sC5b9), for complement activation; and circulating dsDNA, for neutrophil extracellular traps (NETs), were analyzed. The endothelial activation and stress index (EASIX) and C-reactive protein were evaluated.

**Results:**

Before ECP, endothelial damage biomarkers were elevated in all patients, with no significant differences between GR and PR. After 1-month, increased levels of REG3alpha and sC5b9, and decreased levels of TIM3, were observed in samples from PR. A panel combining 5 biomarkers (ST2, VWF, NETs, TIM3, ANG2) could identify GR after 1-month on ECP (likelihood ratio 2.0) and predict ECP response.

**Discussion:**

We propose a simplified endothelial damage biomarker panel capturing early biological signals associated with response to ECP in SR-aGVHD patients.

## Introduction

Allogeneic hematopoietic cell transplantation (allo-HCT) is a widely established treatment for the cure of multiple hematological diseases. However, the procedure carries significant risk, and acute graft-versus-host disease (aGVHD) is a common and potentially life-threatening complication (1, 2). Acute GVHD may appear within the first 100 days after infusion of hematopoietic cells, and apoptosis and cellular necrosis are observed, being the affected organs like the skin, the liver and the digestive tract (1). The pathophysiology of GVHD is complex, involves an attack by donor T cells on recipient target organs, leading to cell damage and apoptosis and causing clinical symptoms. Still, it involves an attack by donor T cells on recipient target organs, leading to cell damage and apoptosis and causing the clinical symptoms of aGVHD (3). Despite the prophylaxis used, aGVHD may appear and corticosteroids are the first-line treatment, based on prospective randomized studies (1, 4). The response to corticosteroid therapy in different retrospective studies is 50% and lack of response is a poor prognostic factor, with an overall survival of 5%-30% (5). Following FDA and EMA approval, ruxolitinib has emerged as the preferred therapy for steroid-refractory acute GVHD (SR-aGVHD). It is now routinely employed as second-line treatment, while extracorporeal photopheresis (ECP) is generally reserved for third-line use in cases of suboptimal response to ruxolitinib or is combined with ruxolitinib in patients with severe disease phenotypes or clinically significant cytopenias (6, 7).

ECP is a therapeutic procedure used in different immunological disorders. After its approval as a treatment for cutaneous T-cell lymphoma in 1988, ECP has been used in the treatment of other T-cell-mediated disorders such as arthritis, rheumatoid arthritis, systemic sclerosis, systemic lupus erythematosus, and GVHD (8–10). ECP consists of the collection of mononuclear cells (MNC) from the patient with an apheresis device, incubation of collected MNCs with 8-methoxypsoralen (8-MOP), and illumination with ultraviolet A (UVA) light. ECP is not immunosuppressive, and it does not exhibit a negative impact on the graft-versus-malignancy effect of the transplant.

The vascular endothelium is an active biological interface between the blood and all other tissues, maintains the blood fluidity, mediates the vasomotor tone and the hemostatic balance, and regulates the permeability of the vessel wall as well as the inflammatory reactions, among other functions (11, 12). Authors have previously demonstrated that endothelium is injured early after allo-HCT by the consecutive effects of the conditioning regimen, the proinflammatory agents used during transplantation, the translocation of endotoxins across the damaged gastrointestinal tract, and the hematopoietic engraftment (13, 14). This research group has also proved that endothelial damage is aggravated in those allo-HCT recipients who develop aGVHD when compared with allo-HCT recipients who do not (15, 16). However, little is known about the endothelial damage in those patients with SR-aGVHD who receive ECP as a second-line therapy.

Therefore, this study aimed to investigate retrospectively the endothelial cell damage in patients with SR-aGVHD who received second-line therapy with ECP, by measuring longitudinally, 11 different endothelial biomarkers.

## Materials and methods

### Experimental design

The present investigation was carried out to study endothelial damage biomarkers in plasma samples from 35 patients with SR-GVHD who initiated ECP treatment as second line therapy. SR-aGVHD patients treated with ECP (n=35) were classified according to their clinical improvement after ECP according to the modified Glucksberg grading system^10^: good responders (GR; n=18) were those who achieved a complete response or partial response; and poor responders (PR; n=17) were those who did not respond. Plasma samples were collected at baseline (PRE) and after 1 month (1-month) of ECP treatment.

The main objective was to longitudinally analyze endothelial activation biomarkers, including soluble Vascular Cell Adhesion Molecule-1 (sVCAM-1), von Willebrand Factor (VWF), thrombomodulin (TM), soluble TNF receptor 1 (sTNFR1), and angiopoietin 2 (ANG2); GVHD-associated biomarkers, such as suppression of tumorigenicity 2 (ST2), regenerating islet-derived 3-alpha (REG3alpha), and T-cell immunoglobulinmucin-3 (TIM3); the complement activation markers soluble C5b9 (sC5b9); and circulating double-stranded DNA (dsDNA) as an indicator of neutrophil extracellular traps (NETs). In addition, the Endothelial Activation and Stress Index (EASIX), and C-reactive protein (CRP) levels were also recorded and evaluated in the study at baseline. Results were compared in each group and between both groups.

### Patients and blood samples

A retrospective study of all adult patients with aGVHD who underwent ECP procedures between October 2012 and December 2021 at the Apheresis and Cellular Therapy Unit at the Hospital Clínic of Barcelona was conducted. The study protocol was reviewed and approved by the Ethics Committee of the Hospital Clínic (HCB/2015/0272). All patients signed an informed consent to receive ECP and consented to data release.

Acute GVHD was diagnosed according to clinical criteria and, in some cases, histopathological criteria (17, 18) and was graded according to the modified Glucksberg grading system. Failure of first-line therapy was defined according to common criteria previously reported for patients with aGVHD (19). All patients received corticosteroids as first-line treatment; they were still treated with corticosteroids at the time of starting (ECP 71% were on a dose of 2 mg/kg/day, and 29% on a dose of 1 mg/kg/day), and along the first month on ECP, during which most of the patients initiated tapering. ECP was performed as reported previously by Cid et al. The median interval between allo-HCT and ECP initiation was 67 days (range, 20–163 days). During the first month of ECP, GR received a median of 7 ECP (range, 5-7) and PR a median of 7 ECP (range, 2-7), and all patients were treated with twice-weekly sessions for the initial two weeks, followed by 1 day per week for the subsequent two weeks (20). Response to ECP was 1 month after starting treatment with ECP. Patients with aGVHD were considered responders to ECP if a complete response or partial response was observed in the grade according to the Glucksberg grading system. During treatment with ECP, twenty patients had an active infection: 85% viral infections (mostly caused by cytomegalovirus), 10% fungal infections, and 5% bacterial infections. In relation to other treatments, nine patients (25.7%) received a third line of treatment during the month under ECP: 3 calcineurin inhibitors, 2 mycophenolate, 2 ruxolitinib, 1 mTOR inhibitor, and 1 mesenchymal stem cell therapy. Three out of these nine patients required a fourth line of treatment (2 mesenchymal stem cells and 1 etanercept) due to the severity of their symptoms, all of whom had gastrointestinal stage III-IV GVHD.

Plasma samples from peripheral veins were collected just before starting treatment with ECP (baseline) and after 1 month receiving treatment with ECP (1-month) and cryopreserved.

### Circulating biomarkers

Plasma levels of sVCAM-1 (ng/mL; Sigma-Aldrich, Madrid, Spain), TM (ng/mL, R&D Systems, Minneapolis, MN, USA), sTNFR1 (pg/mL; R&D Systems), ANG2 (pg/mL; R&D Systems), ST2 (ng/mL; Abcam, Cambridge, United Kingdom), REG3alpha (ng/mL; Abcam), TIM3 (pg/mL; R&D Systems), and sC5b9 (ng/mL; Quidel, San Diego, CA, USA) were measured by ELISA kits. VWF antigen (%; VWF : Ag), and VWF activity (%; VWF : GPIbM) were evaluated in Atellica 360 (COAG, Siemens Healthineers, Germany). Circulating dsDNA (NETs) was quantified using Quant-iT PicoGreen dsDNA Assay Kit (ng/mL; Invitrogen/ThermoFisher Scientific, Waltham, MA, USA). EASIX was retrospectively calculated based on the results of creatinine, LDH and platelets from laboratory routine test, according to the following formula: creatinine (mg/dL) x lactate dehydrogenase (LDH; U/L) / platelets (x 10^9^/L). CRP values were also retrieved from routine laboratory analyses performed at baseline.

### Outcomes, assessments, and statistical analysis

Results from circulating biomarkers were analyzed by using non-parametric tests because of the small sample size (n=35) and non−normally distributed data. Mann-Whitney U test and Wilcoxon test were used to compare two unrelated groups and two related groups, respectively. Student’s t-test was used for paired data with normal distribution Predictive performance of biomarkers to identify individuals who were more likely to experience a response at 1 month after starting ECP was evaluated by measuring the area under the curve (AUC) from the receiver operating characteristic (ROC) curves. Composite panel for ST2, VWF antigen, NETs, TIM3, and ANG2 resulted from a logistic regression, categorizing the independent variable (1 = response and 0 = No response, to ECP after 1 month). Positive and negative predictive values (PPV and NPV) were estimated from data extrapolated from the resulted ROC curves, considering the optimal cutoff (derived from the best likelihood ratio, LR).

Any adverse event (AE) while performing the ECP procedure was recorded. Other AEs were collected after checking medical records of the patient, according to the evaluation of the treating physician.

Qualitative and quantitative data were presented as number (frequencies) and median (interquartile range –IQR-), respectively. Overall survival (OS) was measured from the initiation of ECP using Kaplan-Meier survival curves. We carried out statistical analysis with software (IBM SPSS Statistics 23, IBM Corporation, Armonk, NY).

## Results

### Patients’ characteristics

The main characteristics of the patients are detailed in **Table 1**, according to their response to ECP, measured after 1 month of receiving ECP therapy.

**Table 1:** Main characteristics of patients with steroid-refractory aGVHD (SR-aGVHD) treated with extracorporeal photopheresis (ECP), according to their response at 1-month after starting ECP therapy and classified as good (GR) and poor (PR) responders.

Characteristic*	All patients n=35	ECP GR n=17	ECP PR n=18
Gender			
Male Female	20 (57%) 15 (43%)	10 (59%) 7 (41%)	10 (56%) 8 (44%)
Age (years)†	53 (45-63)	57 (47-63)	52 (43-57)
Weight (Kg)	66 (56-77)	64 (52-75)	68 (58-81)
Total blood volume (mL)	4,429 (3,712-5,116)	4,403 (3,420-5,125)	4,466 (3,787-5,122)
Disease			
Acute leukemia Others	14 (40%) 21 (60%)	6 (35%) 11 (65%)	8 (44%) 10 (56%)
Donor type			
Matched family Haploidentical Matched unrelated	7 (20%) 4 (11%) 24 (69%)	3 (17%) 3 (17%) 11 (64%)	4 (22%) 1 (6%) 13 (72%)
Conditioning regimen			
Myeloablative Non myeloablative	19 (54%) 16 (46%)	8 (47%) 9 (53%)	11 (61%) 7 (39%)
GVHD prophylaxis			
Cyclosporine A Cyclophosphamide Others	7 (20%) 20 (57%) 8 (23%)	2 (12%) 11 (65%) 4 (23%)	5 (28%) 9 (50%) 4 (22%)
CMV serology			
Donor negative / Recipient negative Other combinations	3 (9%) 32 (91%)	3 (18%) 14 (82%)	0 18 (100%)
Gender mismatch			
F donor / M recipient Other combinations	5 (14%) 30 (86%)	4 (23%) 13 (77%)	1 (6%) 17 (94%)
ABO compatibility			
Identical Major Minor Mixed	23 (66%) 4 (11%) 5 (14%) 3 (9%)	14 (82%) 2 (12%) 0 1 (6%)	9 (50%) 2 (11%) 5 (28%) 2 (11%)
Acute GVHD diagnosis (days after stem cell transplant)	43 (28-69)	48 (28-82)	42 (27-67)
Maximum acute GVHD staging			
I II III IV	0 19 (54%) 12 (34%) 4 (12%)	0 12 (71%) 5 (29%) 0	0 7 (39%) 7 (39%) 4 (22%)
ECP start (days after stem cell transplant)	69 (44-110)	77 (43-114)	63 (46-112)
ECP procedures (during the 1^st^ month)	7 (2-7)	7 (5-7)	7 (2-7)

*Data are reported as numbers (frequencies) and median (interquartile range, IQR). †Age when starting ECP. F, Female; M, Male.

Our series comprises 35 patients with aGVHD who were treated with ECP as a second-line therapy. Acute leukemia was the indication for allo-HCT in 40% of patients and the majority, in both groups, received allo-HCT from HLA-identical unrelated donors. Myeloablative conditioning was administered to 11 patients (61%) in group PR and to 8 patients (47%) in group GR and PCTy-based prophylaxis was used in 57% of the patients. Peripheral blood was the predominant stem cell source in the vast majority (97.1%). The maximum severity of GVHD was grade II in 54% of patients, grade III in 34%, and belonging to the PR group 12% with grade IV. Notably, no patient undergoing ECP presented with GVHD below grade II.

### Comparison of circulating biomarkers between responders and non-responders

When comparing circulating biomarkers in baseline samples from GR vs. PR (**Table 2**), no significant differences were observed. However, VWF antigen and VWF activity were significantly elevated in both groups already from the baseline point compared with values within the normal range.

**Table 2:** Circulating biomarkers of baseline samples (before starting extracorporeal photopheresis; ECP) in poor (PR) vs. good (GR) responders.

Biomarkers at baseline	GR after 1-month ECP		PR after 1-month ECP		p value
	n	Median (IQR)	n	Median (IQR)	
sVCAM-1	17	250 (147-279)	18	190 (107-368)	0.9
sTNFR1	17	2729 (2084-3212)	18	2634 (2014-4040)	0.9
TM	17	15 (12-22)	18	17 (9-21)	0.8
VWF antigen	17	286 (255-435)	18	395 (295-440)	0.4
VWF activity	17	289 (223-423)	18	353 (259-461)	0.7
NETs	17	150 (140-160)	18	150 (130-200)	0.6
REG3alpha	17	10 (5-15)	18	8 (5-12)	0.9
ST2	17	42 (33-44)	18	44 (43-46)	0.1
TIM3	17	6027 (4097-10630)	18	6321 (2965-14852)	0.8
ANG2	17	1883 (1109-2433)	18	1292 (567-2430)	0.4
sC5b9	17	686 (586-830)	18	661 (465-899)	0.7
CRP	17	0.72 (0.4-1.9)	18	0.7 (0.4-4)	0.9
EASIX	17	4.1 (2.6-10.5)	18	3.3 (2.1-6.4)	0.6
EASIX log2	17	2 (1.4-3.4)	18	1.7 (1.1-2.7)	0.6

Data are expressed as median (interquartile range, IQR). Results were analyzed by the Mann-Whitney U test. Biomarkers: soluble Vascular Cell Adhesion Molecule-1 (sVCAM-1, ng/mL), soluble TNF receptor 1 (sTNFR1, pg/mL), thrombomodulin (TM, ng/mL), von Willebrand Factor (VWF antigen and activity, %), neutrophil extracellular traps (NETs, ng/mL), regenerating islet-derived 3-alpha (REG3alpha, ng/mL), suppression of tumorigenicity 2 (ST2, ng/mL), T-cell immunoglobulinmucin-3 (TIM3, pg/mL), angiopoietin 2 (ANG2, pg/mL), soluble C5b9 (sC5b9, ng/mL), C-reactive protein (CRP), and Endothelial Activation and Stress Index (EASIX).

In samples after 1 month under ECP (**Table 3**), these biomarkers showed notable differences between the groups of patients. Levels of 9 out of 11 biomarkers were elevated in PR vs. GR (sTNFR1, TM, VWF antigen, VWF activity, NETs, REG3alpha, ST2, ANG2 and sC5b9), although differences were statistically significant only in two of them (REG3alpha, p=0.03, and sC5b9, p=0.01). Contrary to this tendency, sVCAM-1 and TIM3 were reduced in PR vs. GR at the 1-month timepoint, being only statistically significant for TIM3 (p=0.04).

**Table 3:** Circulating biomarkers of samples after 1-month of receiving extracorporeal photopheresis (ECP) in poor (PR) vs. good (GR) responders.

Biomarkers after 1-month	GR after 1-month ECP		PR after 1-month ECP		p value
	n	Median (IQR)	n	Median (IQR)	
sVCAM-1	17	255 (170-333)	16	215 (117-425)	0.8
sTNFR1	17	2912 (2413-4594)	16	4047 (2823-5717)	0.1
TM	17	8 (6-10)	16	10 (8-20)	0.1
VWF antigen	17	259 (231-478)	16	396 (272-483)	0.4
VWF activity	17	288 (203-481)	16	354 (267-477)	0.2
NETs	17	160 (120-230)	16	200 (160-280)	0.2
REG3alpha	17	5 (3-8)	16	10 (7-13)	0.03
ST2	17	43 (41-44)	16	44 (42-45)	0.1
TIM3	17	17525 (13774-20098)	16	10441 (6302-17091)	0.04
ANG2	17	448 (365-934)	16	1339 (387-2548)	0.1
sC5b9	17	390 (309-617)	16	654 (474-1035)	0.01

Data are expressed as median (interquartile range, IQR). Results were analyzed by the Mann-Whitney U test. Biomarkers: soluble Vascular Cell Adhesion Molecule-1 (sVCAM-1, ng/mL), soluble TNF receptor 1 (sTNFR1, pg/mL), thrombomodulin (TM, ng/mL), von Willebrand Factor (VWF antigen and activity, %), neutrophil extracellular traps (NETs, ng/mL), regenerating islet-derived 3-alpha (REG3alpha, ng/mL), suppression of tumorigenicity 2 (ST2, ng/mL), T-cell immunoglobulinmucin-3 (TIM3, pg/mL), angiopoietin 2 (ANG2, pg/mL), soluble C5b9 (sC5b9, ng/mL).

### Evolution of circulating biomarker levels from baseline to one month under ECP

In GR, the evolution of all circulating biomarkers was analyzed in samples from baseline to one month under ECP (**Table 4**). Those biomarkers that showed an increment after one month compared to baseline were sVCAM-1, sTNFR1, NETs, ST2, TIM3, and the most significant difference was observed for TIM3 (p<0.05). In contrast, levels of TM (p<0.05), VWF antigen, VWF activity, ANG2 (p<0.05), and sC5b9 (p<0.05) were decreased after one month compared to baseline samples.

**Table 4:** Evolution of all circulating biomarkers analyzed in samples from baseline to 1-month under ECP in good responders (GR).

Biomarkers	Baseline		1-month		Friedman test
	n	Median (IQR)	n	Median (IQR)	p value
sVCAM-1	17	250 (147-279)	17	255 (170-333)	0.9
sTNFR1	17	2729 (2084-3212)	17	2912 (2413-4594)	0.3
TM	17	15 (12-22)	17	8 (6-10)	<0.05
VWF antigen	17	286 (255-435)	17	259 (231-478)	0.3
VWF activity	17	289 (223-423)	17	288 (203-481)	<0.05
NETs	17	150 (140-160)	17	160 (120-230)	0.8
REG3alpha	17	10 (5-15)	17	5 (3-8)	<0.05
ST2	17	42 (33-44)	17	43 (41-44)	<0.05
TIM3	17	6027 (4097-10630)	17	17525 (13774-20098)	<0.05
ANG2	17	1883 (1109-2433)	17	448 (365-934)	<0.05
sC5b9	17	686 (586-830)	17	390 (309-617)	<0.05
CRP	17	0.72 (0.4-1.9)	NA	NA	NA
EASIX	17	4.1 (2.6-10.5)	NA	NA	NA
EASIX log2	17	2 (1.4-3.4)	NA	NA	NA

Data are expressed as median (interquartile range, IQR). Results were analyzed by the Wilcoxon test. Biomarkers: soluble Vascular Cell Adhesion Molecule-1 (sVCAM-1, ng/mL), soluble TNF receptor 1 (sTNFR1, pg/mL), thrombomodulin (TM, ng/mL), von Willebrand Factor (VWF antigen and activity, %), neutrophil extracellular traps (NETs, ng/mL), regenerating islet-derived 3-alpha (REG3alpha, ng/mL), suppression of tumorigenicity 2 (ST2, ng/mL), T-cell immunoglobulinmucin-3 (TIM3, pg/mL), angiopoietin 2 (ANG2, pg/mL), soluble C5b9 (sC5b9, ng/mL), C-reactive protein (CRP), and Endothelial Activation and Stress Index (EASIX).

In PR, biomarkers following an ascendant trend from baseline to one month under ECP were sVCAM-1, sTNFR1, NETs, ST2 and TIM3, being statistically significant TIM3 (p<0.05). However, the rest of circulating biomarker levels were diminished, being TM, ANG2 and sC5b9 (p<0.05) the more notable (**Table 5**).

**Table 5:** Evolution of all circulating biomarkers analyzed in samples from baseline to one month under ECP in poor responders (PR).

Biomarkers	Baseline		1-month		Friedman test
	n	Median (IQR)	n	Median (IQR)	p value
sVCAM-1	18	190 (107-368)	16	215 (117-425)	0.7
sTNFR1	18	2634 (2014-4040)	16	4047 (2823-5717)	<0.05
TM	18	17 (9-21)	16	10 (8-20)	0.6
VWF antigen	18	395 (295-440)	16	396 (272-483)	0.1
VWF activity	18	353 (259-461)	16	354 (267-477)	0.2
NETs	18	150 (130-200)	16	200 (160-280)	0.7
REG3alpha	18	8 (5-12)	16	10 (7-13)	0.5
ST2	18	44 (43-46)	16	44 (42-45)	0.1
TIM3	18	6321 (2965-14852)	16	10441 (6302-17091)	0.2
ANG2	18	1292 (567-2430)	16	1339 (387-2548)	0.7
sC5b9	18	661 (465-899)	16	654 (474-1035)	0.3
CRP	18	0.7 (0.4-4)	NA	NA	NA
EASIX	18	3.3 (2.1-6.4)	NA	NA	NA
EASIX log2	18	1.7 (1.1-2.7)	NA	NA	NA

Data are expressed as median (interquartile range, IQR). Results were analyzed by the Wilcoxon test. Biomarkers: soluble Vascular Cell Adhesion Molecule-1 (sVCAM-1, ng/mL), soluble TNF receptor 1 (sTNFR1, pg/mL), thrombomodulin (TM, ng/mL), von Willebrand Factor (VWF antigen and activity, %), neutrophil extracellular traps (NETs, ng/mL), regenerating islet-derived 3-alpha (REG3alpha, ng/mL), suppression of tumorigenicity 2 (ST2, ng/mL), T-cell immunoglobulinmucin-3 (TIM3, pg/mL), angiopoietin 2 (ANG2, pg/mL), soluble C5b9 (sC5b9, ng/mL), C-reactive protein (CRP), and Endothelial Activation and Stress Index (EASIX).

### Predictor biomarkers of response at one month after ECP initiation

**Figure 1:**
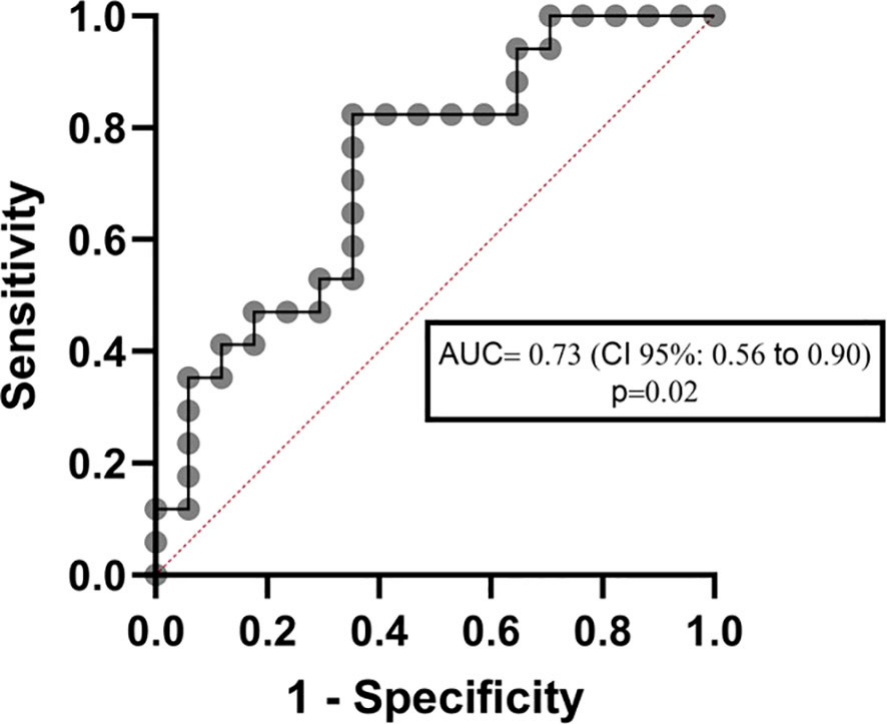
Receiver operating characteristic (ROC) plot for predicting response at 1-month after starting extracorporeal photopheresis (ECP) using a panel consisting of 5 circulating biomarkers (ST2, VWF antigen, NETs, TIM3, and ANG2).

The analysis of the 11 circulating biomarkers, EASIX score and CRP at baseline as predictors of response after 1 month of receiving ECP therapy is shown in **Table 6**. Of note, no single biomarker showed an AUC >0.70. When all these biomarkers are evaluated individually, none of them exhibited sufficient predictive potential. However, when the five biomarkers with AUC >0.55 (ST2, VWF antigen, NETs, TIM3, and ANG2) are integrated into a composite panel, their combined assessment shows a substantially improved ability to predict the response of SR-aGVHD patients to ECP. ROC curve of these five biomarkers presents an AUC = 0.73, CI 95%: 0.56 to 0.90, and p=0.02 (**Figure 1**).

**Table 6:** Predictive performance of biomarkers, to identify individuals who were more likely to respond at 1-month after starting ECP, by measuring the area under the curve (AUC) from the receiver operating characteristic (ROC) curves.

Marker	AUC	CI 95%	p	Cut-off	St (%)	CI 95%	Sp (%)	CI 95%	Likelihood ratio	PPV (%)	NPV (%)
ST2	0.68	0.49 to 0.86	0.07	**>43**	**72.22**	**49.13% to 87.50%**	**70.59**	**46.87% to 86.72%**	**2.456**	71	72
VWF Ag	0.59	0.40 to 0.79	0.33	**>290.7**	**77.78**	**54.79% to 91.00%**	**52.94**	**30.96% to 73.83%**	**1.653**	62	71
NETs	0.58	0.38 to 0.77	0.43	**>136.5**	**72.22**	**49.13% to 87.50%**	**29.41**	**13.28% to 53.13%**	**1.023**	50	51
TIM3	0.56	0.36 to 0.75	0.57	**>5266**	**72.22**	**49.13% to 87.50%**	**47.06**	**26.17% to 69.04%**	**1.364**	58	63
ANG2	0.56	0.36 to 0.76	0.57	**<2433**	**76.47**	**52.74% to 90.44%**	**29.41**	**13.28% to 53.13%**	**1.083**	52	55
TM	0.55	0.36 to 0.74	0.62	<21.6	77.78	54.79% to 91.00%	29.41	13.28% to 53.13%	1.102	52	57
VWF Act	0.55	0.35 to 0.75	0.62	>291.2	72.22	49.13% to 87.50%	58.82	36.01% to 78.39%	1.754	64	68
sTNFR1	0.54	0.34 to 0.74	0.69	>2287	72.22	49.13% to 87.50%	35.29	17.31% to 58.70%	1.116	53	56
sVCAM1	0.54	0.35 to 0.74	0.67	>158.6	72.22	49.13% to 87.50%	41.18	21.61% to 63.99%	1.228	55	59
sC5b9	0.52	0.33 to 0.72	0.80	<917.1	72.22	49.13% to 87.50%	23.53	9.55% to 47.26%	0.944	49	46
REG3A	0.51	0.32 to 0.71	0.91	>5.15	72.22	49.13% to 87.50%	29.41	13.28% to 53.13%	1.023	50	51
CRP	0.54	0.35 to 0.74	0.67	>0.97	50	29.03% to 70.97%	64.71	41.30% to 82.69%	1.417	43	41
EASIX	0.52	0.32 to 0.72	0.84	<2.59	38.89	20.31% to 61.38%	76.47	52.74% to 90.44%	1.653	35	32
Only AUC >0.55	0.73	0.56 to 0.90	0.02	**>0.5**	**70.6**		**64.7**		**2.0**	68	73

St, sensitivity; Sp, specificity; CI, confidence interval; PPV, positive predictive value; NPV, negative predictive value. Biomarkers: soluble Vascular Cell Adhesion Molecule-1 (sVCAM1, ng/mL), soluble TNF receptor 1 (sTNFR1, pg/mL), thrombomodulin (TM, ng/mL), von Willebrand Factor antigen (VWF Ag, %) and von Willebrand Factor activity (VWF Act, %), neutrophil extracellular traps (NETs, ng/mL), regenerating islet-derived 3-alpha (REG3A, ng/mL), suppression of tumorigenicity 2 (ST2, ng/mL), T-cell immunoglobulinmucin-3 (TIM3, pg/mL), angiopoietin 2 (ANG2, pg/mL), soluble C5b9 (sC5b9, ng/mL), C-reactive protein (CRP), and Endothelial Activation and Stress Index (EASIX).

In bold, the five biomarkers with AUC >0.55 that are integrated into a composite panel. Their combined assessment shows a substantially improved ability to predict the response of SR-aGVHD patients to ECP.

## Discussion

**Figure 2:**
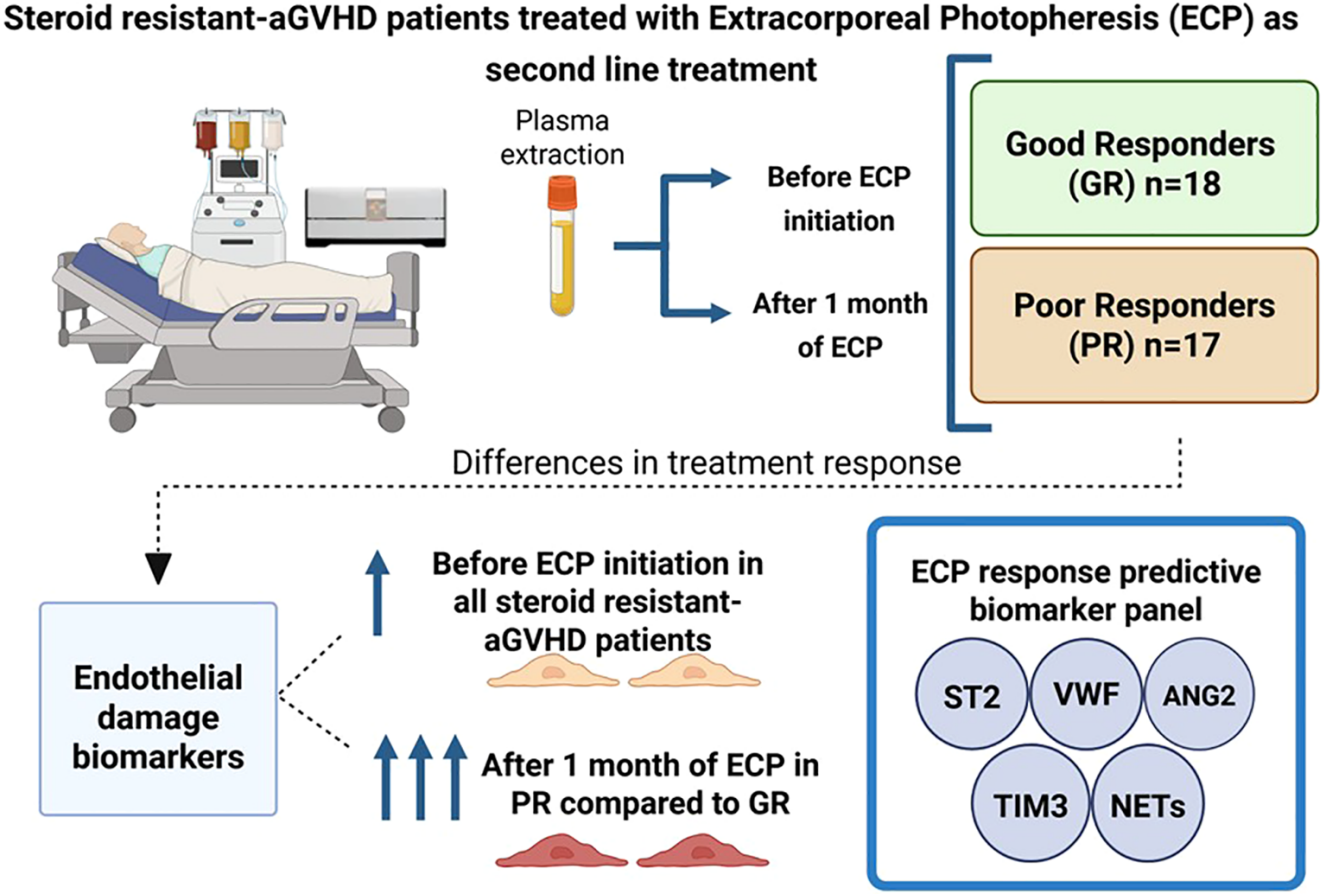
Graphical abstract. A concise and visual summary of the main findings of the present study. The figure illustrates that patients suffering from steroid−refractory acute graft−versus−host disease (SR−aGVHD) presented sustained endothelial damage. Through the present study, the kinetics of markers of endothelial damage were measured in patients under extracorporeal photopheresis (ECP) as second−line therapy, to distinguish between good (GR) and poor (PR) responders after one month on ECP treatment. PR exhibited higher levels of most of the biomarkers measured compared to GR after one month on ECP. Levels of a panel of five plasma biomarkers (ST2, VWF, ANG2, TIM3, NETs) before ECP showed predictive value of ECP response at one month.

Through the present study, we observed biomarker patterns suggestive of persistent endothelial damage in patients with SR-aGVHD, which remains detectable during ECP used as second-line therapy. Furthermore, a panel of five plasma biomarkers measured before ECP initiation was able to explore the dynamics of these markers associated with clinical response at one month (**Figure 2**).

Increasing evidence suggests that endothelial cell (EC) activation and dysfunction occur in association with allo-HCT and are early and critical events in the complications that appear early, such as veno-occlusive disease / sinusoidal obstruction syndrome (VOD/SOS), transplantation associated thrombotic microangiopathy (TA-TMA) and GVHD (2, 21–23). In GVHD, the endothelial interface not only reflects systemic inflammatory stress but also contributes to the amplification of immune-mediated tissue damage (15, 23, 24).

A substantial number of clinical and preclinical studies support the relevance of EC activation markers such as VCAM-1, ICAM-1, VWF : Ag, TNFR1, E-selectin, ANG2, and soluble TM to demonstrate endothelial damage in GVHD, some of them being able to predict its development (15, 25).

Our findings support and contribute the current understanding of endothelial dysfunction as an important mechanism in aGVHD and its modulation by ECP. In our cohort, patients with SR-aGVHD treated with ECP, it was observed that levels of endothelial activation markers such as VWF : Ag and VWF activity were already markedly elevated at baseline in both good and poor responders, proposing preexisting endothelial stress before ECP initiation. Neidemire-Colley et al. provided a comprehensive mechanistic review of how ECs respond to allo-HCT-associated insults (including conditioning regimens, cytokine storms, and direct T-cell-mediated cytotoxicity) resulting in loss of junctional integrity and barrier function in target organs such as the gut and liver. Notably, reductions in VE-cadherin and ZO-1 expression were associated with increased vascular permeability and tissue infiltration by alloreactive T cells. This work highlighted the pivotal role of adhesion molecules (selectins, integrins) and Weibel-Palade body-derived factors (e.g., VWF, P-selectin) in leukocyte-endothelium interactions that propagate GVHD (26).

Clinical relevance of these findings is reinforced by Luft et al. who demonstrated that the EASIX serves as a strong prognostic marker after GVHD, accurately predicting outcomes in this population of patients (27–29). Pedraza et al. also highlighted the combination of TNFR1 ≥1300 ng/mL and log2-EASIX ≥3 in the immediate post-HCT phase displayed a robust predictive value for the development of aGVHD. Levels of sVCAM-1, VWF: Ag, and sTNFR1 on day +7 post-HCT were independently predictive of aGVHD (30). The findings of our work are consistent with prior reports by Mir et al. and Pedraza et al., who identified elevated VWF as a key feature of endothelial injury in early GVHD (15, 30).

Although steroids are the gold standard for the treatment of aGVHD (4, 31), a high proportion of patients (20%-25%) fail to respond, exhibiting very high mortality (32, 33). Despite FDA and EMA approval and the European Bone and Marrow Transplantation recommendations supporting ruxolitinib, toxicity or lack of efficacy in certain patients necessitates the search of alternative approaches. Nonetheless, in Europe, no standard treatment for steroid-refractory aGVHD beyond ruxolitinib is currently available (31), and its pathobiology is poorly understood, but there is evidence associating endothelial damage and steroid refractoriness (25, 34, 35). In human biopsies and murine tissues from SR-aGVHD individuals, there was extensive tissue damage with low levels of alloreactive T cell infiltration in target organs. These results support the rationale for T cell-independent SR-aGVHD treatment strategies, specifically considering endothelial damage as a target (23). In addition, by using a proteomic approach to compare the sera of steroid-refractory and non-refractory aGVHD patients, our group has recently demonstrated that there is a protein signature characteristic for steroid-refractoriness (36). Some of the proteins identified could constitute potential biomarkers of response to steroid treatment.

ECP appears as an alternative for the treatment of steroid-refractory aGVHD patients (7). In earlier clinical observations, Cid et al. reported a one-day ECP protocol yielding a 57% response in aGVHD, reinforcing the practical feasibility and real-world efficacy of ECP (20) but its impact on the endothelium has not been explored until now. In the present study, after one month of ECP treatment, a divergent biomarker profile emerged between good and poor responders' patients. Notably, 9 of the 11 biomarkers analyzed increased in poor-responders, suggesting persistent or exacerbated endothelial damage. Among these, REG3alpha and sC5b9 were significantly elevated, indicating ongoing epithelial and complement activation, respectively. In contrast, good responders' patients showed significant reductions in TM, ANG2, sC5b9, and a decreasing trend in VWF : Ag and VWF activity, supporting a recovery of endothelial stability following ECP. These changes mirror the protective endothelial effects previously described by Martinez-Sanchez et al. and Hagn et al., who reported similar reductions in EC activation markers and inflammatory mediators in experimental models following defibrotide or ECP (16, 37). Interestingly, TIM3 emerged as a differentially expressed molecule in both groups at the one-month timepoint. Its significant decrease in poor responders compared to good responders (p=0.04), and its dynamic regulation over time, suggests a potential role in endothelial-immune crosstalk, though further mechanistic studies are warranted.

To further explore the prognostic utility of these biomarkers, we constructed a predictive panel composed of five markers (ST2, VWF : Ag, NETs, TIM3, and ANG2), which achieved an AUC of 0.73 (p=0.001), with a PPV and NPV of 68% and 73%, respectively. This is particularly relevant given that none of the individual markers exceeded an AUC of 0.70 when evaluated alone. These results resonate with the work of Bojanic et al. and Mankarious et al., who proposed multi-marker approaches, including microRNAs and EMPs, as more robust tools for capturing the complexity of endothelial activation and therapeutic response (38, 39).

Taking all together, our data support the concept that, in responsive patients, ECP exerts an immunomodulatory effect that may decrease the endothelial damage, as shown by the reduction in endothelium activation markers. The observed endothelial damage biomarker dynamics may reflect a shift from a pro-inflammatory endothelial state toward a stabilized vascular phenotype. These findings might offer new insights into the biological changes of ECP efficacy and suggest that endothelial biomarker panels may be useful tools for early response stratification in aGVHD.

This study has several limitations that should be considered when interpreting our results. First, its retrospective design may introduce selection bias and unmeasured confounding. Second, the relatively small sample size from a single institution limits the generalizability of our findings. In addition, patients diagnosed with aGVHD over a prolonged period (2012-2021), during which classification and grading criteria, and transplant protocols evolved. Although we applied standardized scales to harmonize the population, some degree of heterogeneity may persist. Moreover, treatment outcomes may have been influenced by concomitant therapies, such as corticosteroid treatment itself, and variations in clinical management, which were not fully controlled in this analysis.

Overall, these results should be considered exploratory and hypothesis−generating. Future prospective studies using a larger cohort of ECP patients, with samples collected on a weekly basis, and validated criteria, such as the MAGIC classification, are needed to confirm these findings and to better define the predictive value of these biomarkers for treatment response and relapse-free mortality over time.

## Data Availability

The raw data supporting the conclusions of this article will be made available by the authors, without undue reservation.
